# A Retrospective Cohort Study of the Impact of Implementing Volume-Targeted Compared to Pressure-Limited Ventilation in a Single-Center, Level III Neonatal Intensive Care Unit in Oman

**DOI:** 10.7759/cureus.55731

**Published:** 2024-03-07

**Authors:** Hilal Al Mandhari, Zainab Al Maawali, Hilal Al Saadi, Ashfaq Khan

**Affiliations:** 1 Neonatal Unit, Child Health Department, Sultan Qaboos University Hospital/Sultan Qaboos University, Muscat, OMN; 2 College of Medicine and Health Sciences, Sultan Qaboos University, Muscat, OMN; 3 Pediatrics, Oman Medical Specialty Board, Muscat, OMN

**Keywords:** neonate, tidal volume, pressure-controlled ventilation volume guaranteed, pressure-controlled ventilation, artificial ventilation

## Abstract

Background

The use of volume-targeted ventilation (VTV) in neonatology has been introduced in the last decade. This study was performed to determine the impact of clinical implementation of volume-targeted conventional mechanical ventilation using the volume guarantee mode in mechanical ventilation of all neonates needing mechanical ventilation compared to pressure-limited ventilation (PLV) modes. The mortality rate, duration of mechanical ventilation, and bronchopulmonary dysplasia were the primary outcomes of the study.

Methodology

This retrospective cohort study was conducted at a level III-VI neonatal intensive care unit (NICU) within a tertiary academic hospital in Oman. All intubated neonates admitted to the NICU within two time periods, i.e., the PLV cohort: January 2011 to December 2013 (three years), and the VTV cohort: January 2017 to December 2019 (three years), were eligible for inclusion in the study. Neonates were excluded if they had multiple congenital anomalies, tracheostomy, and those with a Do Not Resuscitate status. A predetermined data set was collected retrospectively from electronic records. The PLV and VTV cohorts were compared, and SPSS version 25 (IBM Corp., Armonk, NY, USA) was used for data analysis.

Results

A total of 290 neonates were included (PLV: n = 138, and VTV: n = 152). The two cohorts were statistically similar in their baseline characteristics, including gestational age, birth weight, Apgar scores, indications for mechanical ventilation, age at intubation, need for surfactant therapy, and age at extubation. The VTV cohort had a significantly lower mortality rate (n (%) = 10 (6.6%) vs. 21 (15.3%), p = 0.02). An insignificant trend of lower duration of ventilation was observed in the VTV cohort (34.5 vs. 50.5 hours, p = 0.24). There was no significant difference in bronchopulmonary dysplasia (16 (21.3%) vs. 12 (17.8%), p = 0.18). VTV was associated with a significant reduction in pulmonary hemorrhage (1 (0.7%) vs. 8 (5.7%), p = 0.04), episodes of hypocapnia (2 vs. 3/patient, p = 0.04), and episodes of hypercapnia (0 vs 1/patient, p = 0.04).

Conclusions

The implementation of VTV in clinical practice in our level III-VI NICU was associated with significant advantages, including reduction in mortality, pulmonary hemorrhage, and episodes of hypercapnia and hypocapnia. A large prospective, randomized, and multicenter trial is recommended to confirm these findings.

## Introduction

Mechanical ventilation is one of the most important innovations that have improved the survival of patients with various causes of respiratory failure, especially preterm infants with respiratory distress syndrome (RDS) [1]. In the United States, for every 1,000 live newborns, 18 infants require mechanical ventilation, with the highest rate among infants weighing less than 800 g at birth [2]. Historically, the intermittent mandatory ventilation (IMV) mode was used in the mechanical ventilation of neonates. In this mode of mechanical ventilation, pressure-controlled scheduled ventilator (mandatory) breaths occur between spontaneous breaths. However, due to patient-ventilator asynchrony and poor interaction between the ventilator and spontaneous breathing, many complications were observed, including gas trapping, inadequate gas exchange, air leaks, and fluctuation in intracranial pressure [3]. After 1990, synchronized intermittent mandatory ventilation (SIMV) became the standard mode of mechanical ventilation in which mandatory breaths are patient-triggered. Patient-triggered ventilation has advantages such as improved ventilation, fewer complications, more consistent ventilator pressures, and improved patient comfort, thus facilitating weaning [4].

One form of patient-triggered ventilation is pressure-limited ventilation (PLV) which may be used alone or in combination with SIMV. In PLV modes, each cycle has a preset peak inspiratory pressure (PIP) that makes the tidal volume (VT) delivery vary according to lung compliance [5]. Administration of surfactant-replacement therapy causes enhancement of lung compliance, which may lead to excessive VT delivery. This, in turn, may cause ventilator-induced lung injury due to volutrauma, overexpansion, and inflammation [6]. Such lung injury may, in turn, trigger inflammatory responses that ultimately contribute to the evolution of bronchopulmonary dysplasia (BPD) [5].

Recently, a new modality has been used to cover the deficiencies of PLV, volume-targeted ventilation (VTV). VTV is not a new concept, but due to technology limitations in the past, it was not used. However, with advances in technology, the use of such a modality is now possible. In VTV modes, the PIP is automatically adjusted depending on the lungs’ compliance, so the VT delivery is maintained constant [7]. VTV use is associated with a shorter duration of mechanical ventilation [5,8,9]. A recent Cochrane review of 20 studies revealed lower combined mortality and BPD rates in neonates who received VTV compared to PLV [10].

There are so far no published studies comparing the clinical effects of using VTV to PLV from the Middle Eastern region. This study was conducted at a level III-IV neonatal intensive care unit (NICU) aiming to determine the impact of implementing routine use of the VTV mode and compare it to an older period of using PLV modes. The study’s main objective was to compare the mortality rate, BPD, and duration of invasive ventilation during the VTV era compared to the PLV era.

## Materials and methods

Study settings

This study was conducted in a university-based, level III-IV neonatal unit in Oman located within a tertiary academic hospital with an average of 4,000 deliveries/year. Before 2014, all intubated neonates were mechanically ventilated using pressure-limited or pressure-control ventilation modes (e.g., pressure control assist control (PCAC), SIMV, and synchronized mandatory positive pressure ventilation). With increasing evidence of the benefits of VTV, in 2014, the team at our NICU switched to the routine use of VTV using PCAC+ volume guarantee (VG) mode. Neonates were ventilated using Draeger Babylog® VN500 ventilators. In our NICU, blood gas analysis is performed routinely in all intubated neonates every 6, 8, or 12 hours (depending on clinical stability) and as necessary, especially when changes are made to VT in the VTV cohort or PIP in the PLV cohort. Based on the clinical condition of the patients and/or changes made to the ventilatory parameters, the weaning of invasive mechanical ventilation is a physician-dependent approach. Ventilation parameters are weaned when the overall condition has improved, partial pressure of carbon dioxide (pCO_2_), and improved oxygenation. Generally, in the PLV cohort, the weaning of PIP was by 2 cmH_2_O, and in the VTV cohort, the VT was weaned by 0.5 cmH_2_O until reaching a minimum of 4 mL/kg. Then, the respiratory rate (RR) is weaned by five points until reaching a minimum of 30 breaths per minute. Ventilated infants are generally extubated to non-invasive respiratory support; either continuous positive airway pressure, non-invasive positive pressure ventilation, or high-flow nasal cannula based on the baby’s size. However, physician discretion also contributes to the type of post-extubation respiratory support.

Study design and population

This study is a retrospective cohort study. All intubated infants admitted to the NICU within two time periods (PLV cohort: January 2011 to December 2013 and VTV cohort: January 2017 to December 2019) were eligible for inclusion in the study. However, neonates with multiple congenital anomalies, tracheostomy, and those with Do Not Resuscitate status were excluded. The reason for excluding the period between 2014 and 2016 was to avoid the transitional period of introducing VTV in clinical practice. Ethical approval was obtained from the institutional Medical Research Ethics Committee (MREC) in June 2020 (approval number: MREC #2177). Data were extracted from the patient’s electronic charts.

Data collection

A pre-specified data set was collected for each patient, including gestational age (GA), birth weight (BW), Apgar score at one and five minutes, day of life (DOL) at intubation, DOL at extubation, the reason for mechanical ventilation, duration of invasive mechanical ventilation in hours, extubation outcome: success or failure (EF, defined as the need of re-intubation within seven days of extubation), number of extubation failures if any, number of episodes of hypocapnia (pCO_2_ <35 mmHg), number of episodes of hypercapnia (pCO_2_ >55 mmHg), pneumothorax, the total number of blood gas analyses during the invasive mechanical ventilation period, ventilation parameters before extubation (including positive end-expiratory pressure (PEEP), RR, fractional inspired oxygen concentration (FiO_2_), and type of post-extubation respiratory support. For preterm infants, additional information was collected, including surfactant use, pulmonary hemorrhage, BPD, patent ductus arteriosus, interventricular hemorrhage (IVH), and the use of dexamethasone for extubation. BPD was defined based on the recent update of the National Institutes of Child Health and Human Development’s workshop definition as the continued need for oxygen therapy or respiratory support at the postmenstrual age of 36 weeks or discharge, whichever comes first [11]. BPD as an outcome in this study was only investigated in infants born at less than 32 weeks gestational age.

Study outcomes

The primary outcomes of this study were mortality rate, duration of mechanical ventilation (in hours), and the rate of BPD in preterm infants <32 weeks. The number of episodes of hypocapnia and hypercapnia, the number of blood gases performed during the mechanical ventilation period, pulmonary hemorrhage, pneumothorax, extubation failure, IVH, and length of stay (days) were secondary outcomes.

Statistical analysis

Statistical analysis was conducted using SPSS version 25 (IBM Corp., Armonk, NY, USA). Descriptive statistics such as mean ± standard deviation (SD) or median and interquartile range (IQR) were calculated for continuous variables. Counts and percentages were calculated for categorized variables. Kolmogorov-Smirnov test was used to test the normality of distribution for continuous variables. The differences in patients’ characteristics between the two cohorts were tested using the two-sample t-test for normally distributed continuous variables, while the Mann-Whitney U-test was used for non-normally distributed continuous variables and the chi-square test was used for categorized variables. A p-value of less than 0.05 was considered statistically significant.

## Results

Patients characteristics

A total of 185 neonates met the initial eligibility for the PLV cohort, of whom 138 were included, and 47 were excluded. For the VTV cohort, a total of 214 neonates met the initial eligibility for the study, of whom 152 were included, and 62 were excluded. The median GA was 30 weeks and 31 weeks for the PLV and VTV cohorts, respectively (p = 0.05). The reasons for intubation were similar in both cohorts, with RDS being the most common reason for intubation in both cohorts (p = 0.131) (Table 1). The intubation occurred on DOL 1 in both study cohorts. However, extubation was performed at a median of DOL 4 in the PLV cohort compared to a median of DOL 3 in the VTV cohort. The pre-extubation FiO_2_ was similar, but pre-extubation PEEP and RR were slightly higher in the VTV cohort. No significant difference was observed in post-extubation respiratory support.

**Table 1:** Baseline characteristics of the patients in the PLV and VTV cohorts. *: Variable expressed as median (interquartile range). ^a^: Mann-Whitney U-test. ^b^: Chi-square test. For the difference between the PLV and VTV cohorts, a p-value <0.05 is significant. CPAP: continuous positive airway pressure; GA: gestational age; BW: birth weight; DOL: day of life; RDS: respiratory distress syndrome; PDA: patent ductus arteriosus; PEEP: positive end-expiratory pressures; FiO_2_: Fractional inspired oxygen; NIV: non-invasive ventilation; PLV: pressure-limited ventilation; VTV: volume-targeted ventilation

Variables		PLV (n = 138)	VTV (n = 152)	P-value
GA (weeks) ^*^		30 (8)	31 (9)	0.05^a^
BW (g)^*^		1,310 (1,423)	1,600 (1,500)	0.11^a^
APGAR 1^*^		5 (3)	6 (4)	0.46^a^
APGAR 5^*^		8 (2)	8 (2)	0.71^a^
Reason for mechanical ventilation, n (%)	RDS	113 (81.9)	115 (76.3)	0.09^b^
	Sepsis	6 (4.3)	2 (1.3)	
	Apnea	6 (4.3)	11 (7.2)	
	Others	13 (9.4)	23 (15.1)	
DOL of intubation^*^		1 (0)	1 (1)	0.20^a^
Surfactant use, n (%)		88 (63.8)	96 (63.2)	0.30^b^
PDA, n (%)		18 (23.3)	31 (27.4)	0.08^b^
Dexamethasone use, n (%)		6 (5.6)	14 (10.3)	0.08^b^
DOL of extubation^*^		4 (4)	3 (4)	0.37^a^
PEEP at extubation (cmH_2_O)^ *^		5 (1)	6 (0)	<0.01^a^
FiO_2_ at extubation ^*^		0.22 (0.09)	0.23 (0.09)	0.80^a^
Respiratory rate at extubation^*^		28 (6)	30 (10)	<0.01^a^
Post-extubation support, n (%)	NIV/CPAP	106 (76.8)	119 (76.9)	0.08^b^
	Nasal cannula	5 (3.6)	17 (11.2)	
	Room air	6 (4.3)	6 (3.9)	

Primary outcomes

A significantly lower mortality rate was observed in the VTV cohort compared to the PLV cohort (VTV vs. PLV: 10 (6.6%) vs. 21 (15.3%), p = 0.02) (Table 2).

**Table 2:** Comparison of primary outcomes in the study cohorts. For the difference between the PLV and VTV cohorts, a p-value <0.05 is significant. BPD: bronchopulmonary dysplasia; IQR: interquartile range; PLV: pressure-limited ventilation; VTV: volume-targeted ventilation

	PLV (n = 138)	VTV (n = 152)	P-value
Mortality, n (%)	21 (15.3)	10 (6.6)	0.02
Duration of mechanical ventilation (hours), median (IQR)	50.5 (127)	34.5 (122.25)	0.24
BPD in infants <32 weeks, n (%)	12 (17.8)	16 (21.3)	0.18

Further analysis of the causes of mortality in the study is shown in Table 3. There was a notable trend of lower deaths due to prematurity and its complications in the VTV cohort (2 (20%) vs. 12 (57.1%)). Sepsis was the cause of death in 5 (50%) of the VTV cohort and only 2 (9.5%) of the PLV cohort.

**Table 3:** Comparison of causes for death in the PLV and VTV cohorts. For the difference between the PLV and VTV cohorts, a p-value <0.05 is significant. PLV: pressure-limited ventilation; VTV: volume-targeted ventilation

	PLV (N = 21), n (%)	VTV (N = 10), n (%)	P-value
Prematurity and its complications	12 (57.1)	2 (20)	0.003
Sepsis	2 (9.5)	5 (50)	
Perinatal asphyxia	0 (0)	2 (20)	
Others	7 (33.3)	1 (10)	
Total	21(100)	10 (100)	

Although the median duration of mechanical ventilation was shorter in the VTV cohort (34.5 hours compared to 50.5 hours in PLV), the difference was statistically not significant (p = 0.24). In addition, there was no significant difference in the rate of BPD in both cohorts (16 (21.3%), vs. 12 (17.8%), p = 0.18) (Table 2).

Secondary outcomes

Significantly lower episodes of hypocapnia, hypercapnia, and pulmonary hemorrhage were observed in the VTV cohort (Table 4). Of note, four cases of mortality in the PLV cohort were related to pulmonary hemorrhage compared to only one case in the VTV cohort. Although the median number of blood gas analyses in the VTV cohort was lower (9 vs. 12), the difference was statistically insignificant. There were no significant differences in other secondary outcomes, including pneumothorax, need for rescue high-frequency oscillatory ventilation, extubation failure rate, IVH, and median length of stay (Table 4).

**Table 4:** Secondary outcomes. *: Variable expressed as median (interquartile range). For the difference between the PLV and VTV cohorts, a p-value <0.05 is significant. HFVO: high-frequency oscillatory ventilation; IMV: invasive mechanical ventilation; IVH: intraventricular hemorrhage; PLV: pressure-limited ventilation; VLV: volume-targeted ventilation

Variables	PLV (n = 138)	VTV (n = 152)	P-value
Number of episodes of hypocapnia/patient^*^	3 (6)	2 (4)	0.04
Number of episodes of hypercapnia/patient^*^	1 (3)	0 (3)	0.04
Number of blood gases/patient during IMV^*^	12 (24)	9 (24)	0.82
IVH, n (%)	20 (18.2)	19 (16.8)	0.40
Pneumothorax, n (%)	15 (11)	14 (9.2)	0.50
Pulmonary hemorrhage, n (%)	8 (5.7)	1 (0.7)	0.04
Need for HFOV, n (%)	24 (17.5)	28 (18.5)	0.82
Extubation failure, n (%)	7 (6)	10 (7)	0.73
Length of stay (days)^*^	31 (42)	28 (46)	0.98

## Discussion

Due to the increasing evidence of the advantages of using VTV in neonates, especially preterm infants, our neonatal team implemented routine use of VTV (using PCAC+VG) when invasive mechanical ventilation is started which is continued during the weaning phase until extubation. This retrospective cohort study investigated the impact of this change in the practice compared to the previous practice of using PLV modes. The study demonstrated evidence indicating the superiority of VTV to PLV, as supported by the findings of a significant reduction in mortality rate, pulmonary hemorrhage, and decrease in the episodes of hypocapnia and hypercapnia in the VTV cohort. Some of these findings are novel to this study and others are supported by previously published literature. To our knowledge, a significant reduction in mortality with the use of VTV has not been reported before. However, trends of lower mortality have been previously described. For instance, in the study by Duman et al. (2012), there was a notable lower mortality in the volume guarantee (VG) group (13% vs. 32%). However, the difference was not significant, probably because their study had a much smaller population [8]. Multiple individual trials showed trends of lower mortality in VTV-ventilated neonates. However, the difference in mortality in each trial was not significant because all had small sample sizes [5,8,12-14]. A detailed look into the causes of death (Table 4) demonstrated a notably higher number of deaths from prematurity complications in the PLV cohort.

There was no significant difference in the rates of BPD between the VTV and PLV cohorts in this study. There have been inconsistent reports regarding the impact of VTV on the rate of BPD. Most previously published trials that compared VTV to PLV modes showed no significant difference in BPD [5,8,13,15-17]. However, the trial by Guven et al. (2013) reported a significant reduction in BPD at 36 weeks of postmenstrual age in the VTV group [13]. BPD is a multifactorial condition with a variety of risk factors, including lower GA and birth weight and exposure to prolonged mechanical ventilation and oxygen [18]. Although the VTV cohort had a trend of shorter duration of mechanical ventilation, this was statistically not significant. The improvement in survival may also explain the trend of the slightly higher BPD rate in the VTV cohort.

The significant decrease in episodes of hypocapnia and hypercapnia is likely related to more stable TV in VTV modes [19]. A systematic review of VTV vs. PLV showed a significantly lower occurrence of hypocapnia [10]. Chen et al. (2019), in a similar study implementing VTV, showed significantly lower episodes of hypercapnia in the VTV group [20].

In this study, a significant decrease in pulmonary hemorrhage was observed in the VTV cohort. We believe that this is a novel finding of this study, as no previous studies have reported this finding. The expected reduction in the risk of barotrauma with the use of VTV may explain this. A similar study by Chen et al. (2019) showed trends of a lower rate of pulmonary hemorrhage in the VTV group (8% vs. 14%); however, the difference was found to be statistically insignificant [20], probably because the population size was much smaller compared to our study. Pulmonary hemorrhage is a well-described prematurity-related complication in preterm neonates with RDS, which is associated with a high mortality rate [21]. An important observation to be noted is the difference in mortality cases due to prematurity complications, which are much higher in the PLV cohort (12 vs. 2 cases), and the significantly high rate of pulmonary hemorrhage in the PLV cohort. We believe that the significant decrease in pulmonary hemorrhage in the VTV cohort has likely contributed to the notable decrease in mortality due to prematurity complications.

The strengths of this study are related to its relatively larger population and the inclusion of a wild range of gestational ages from extreme preterm to term neonates (22-40 weeks) compared to other studies that had been previously reported. Another strength is the novelty of finding a significantly lower mortality rate and pulmonary hemorrhage in the VTV cohort. However, there are limitations to this study that are worth discussing. The retrospective cohort study design has limitations due to the risk of bias related to unmeasured confounding factors. Retrospective studies may encounter issues with data accuracy and consistency, affecting the reliability of the findings. In addition, the study includes two cohorts of neonates from two different periods. As such, changes and developments in clinical practice over time may have contributed to the decrease in the mortality of preterm infants. In addition, the study represents a single-center experience, which may impose limitations on its generalizability. Therefore, future large randomized multicenter studies are recommended to help ascertain the findings of this study.

## Conclusions

The implementation of VTV in our level III-VI NICU was associated with a significant decrease in mortality rate, pulmonary hemorrhage, and episodes of hypocapnia and hypercapnia. The duration of mechanical ventilation and the rate of BPD were statistically unchanged.

Based on the results of this study, the implementation of VTV in clinical practice for neonates who need mechanical ventilation has advantages in patients’ outcomes, and its implementation may be considered to improve patients’ outcomes. However, considering the limitations of retrospective studies, a large prospective randomized, multicenter trial is recommended to confirm these findings.
